# Intact ketogenesis predicted reduced risk of moderate-severe metabolic-associated fatty liver disease assessed by liver transient elastography in newly diagnosed type 2 diabetes

**DOI:** 10.3389/fendo.2023.1306134

**Published:** 2024-01-08

**Authors:** Sejeong Lee, Jaehyun Bae, Seung Up Kim, Minyoung Lee, Yong-ho Lee, Eun Seok Kang, Bong-Soo Cha, Byung-Wan Lee

**Affiliations:** ^1^ Division of Endocrinology and Metabolism, Department of Internal Medicine, CHA Gangnam Medical Center, CHA University School of Medicine, Seoul, Republic of Korea; ^2^ Division of Endocrinology and Metabolism, Department of Internal Medicine, Catholic Kwandong University College of Medicine, International St. Mary’s Hospital, Incheon, Republic of Korea; ^3^ Department of Internal Medicine, Yonsei University College of Medicine, Seoul, Republic of Korea; ^4^ Yonsei Liver Center, Severance Hospital, Seoul, Republic of Korea; ^5^ Division of Endocrinology and Metabolism, Department of Internal Medicine, Yonsei University College of Medicine, Seoul, Republic of Korea

**Keywords:** ketogenesis, β-hydroxybutyrate, steatosis, MAFLD, diabetes

## Abstract

**Aim:**

Hepatic ketogenesis is a key metabolic pathway that regulates energy homeostasis. Some related controversies exist regarding the pathogenesis of metabolic-associated fatty liver disease (MAFLD). We aimed to investigate whether intact ketogenic capacity could reduce the risk of MAFLD based on transient electrography (TE) in patients with newly diagnosed type 2 diabetes (T2D).

**Methods:**

A total of 361 subjects with newly diagnosed T2D were recruited and classified into two groups based on the median serum β-hydroxybutyrate (βHB) level, referred to as the intact and impaired ketogenesis groups. The glucometabolic relevance of ketogenic capacity and associations of the baseline serum β-HB and MAFLD assessed with TE were investigated.

**Results:**

Compared to the impaired ketogenesis group, the intact ketogenesis group showed better insulin sensitivity, lower serum triglyceride levels, and higher glycated hemoglobin levels. The controlled attenuation parameter (CAP) was lower in the intact ketogenesis group without statistical significance (289.7 ± 52.1 vs. 294.5 ± 43.6; p=0.342) but the prevalence of moderate–severe steatosis defined by CAP ≥260 dB/m was significantly lower in the intact group. Moreover, intact ketogenesis was significantly associated with a lower risk of moderate–severe MAFLD after adjusting for potential confounders (adjusted odds ratio 0.55, 95% confidence interval 0.30–0.98; p=0.044).

**Conclusion:**

In drug-naïve, newly diagnosed T2D patients, intact ketogenesis predicted a lower risk of moderate–severe MAFLD assessed by TE.

## Introduction

With pathophysiologic homeostasis of fatty acids (FAs), nonalcoholic fatty liver disease (NAFLD) develops due to the accumulation of overwhelmingly delivered FAs from peripheral tissues or *de novo* lipogenesis and the limited capacity of FA disposal in a milieu of metabolic disorders with no evidence of secondary causes (1). With advances in the understating of disease unmet requirements (2–4), growing interests and studies have accumulated regarding the recently proposed definition of metabolic-associated fatty liver disease (MAFLD) to achieve a more accurate representation of the underlying pathophysiology of fatty liver disease as metabolically driven (5).

Unlike pathological ketogenesis that mainly arises from acute hyperglycemic complications of diabetes in the absence or absolute deficiency of insulin, physiologic ketone bodies are produced under a fasting state, are utilized as compensatory energy sources for extrahepatic tissues, especially for the brain, and are considered to be related to beneficial metabolic effects, according to recent studies (6–8). In stable homeostatic states, ketogenesis could serve as an optimal pathway for the clearance of excess lipids, but it is often impaired and underutilized in specific subgroups of individuals (9–11). In the healthy liver with normal glucose tolerance status, FAs delivered into the liver are disposed of through β-oxidation resulting in the generation of ketone bodies, including acetone, acetoacetate, and β-hydroxybutyrate (βHB), along with down-regulation of *de novo* lipogenesis (6). However, in the metabolic dysregulated liver, FAs driven from peripheral tissues might be oxidized to carbon dioxide via the tricarboxylic acid (TCA) cycle, which promotes gluconeogenesis and increases plasma glucose concentrations (12, 13). Based on previous studies that looked at suppressed ketogenesis in individuals with fatty liver disease (14, 15) and the correlation between ketone bodies and anti-hepatic steatotic effects (16, 17), we recently demonstrated that intact ketogenesis might be associated with decreased risk of hepatic steatosis using non-image based specific MAFLD indices, including NAFLD liver fat scores, Framingham steatosis indices, and other measurements, in patients with newly diagnosed type 2 diabetes (T2D) (18). In the present study, we aimed to define MAFLD more accurately using transient elastography (TE), which is a more reliable method for assessment of the liver steatosis stage to elucidate whether the presence of ketogenesis influences hepatic steatosis in newly diagnosed T2D patients.

## Materials and methods

### Study design and population

From the prospective cohort diabetes registry at the Severance Diabetes Center, a tertiary care hospital in Seoul, which was conducted on newly diagnosed glucose intolerance or diabetic patients, we evaluated the association between ketogenesis and risk of hepatic steatosis in patients with newly diagnosed T2D using a cross-sectional design. The cohort comprised patients who underwent a standardized mixed-meal stimulation test on their first visit to the diabetes center in 2009. The registry protocol required routine collection of blood samples at 0 and 90 minutes (basal and stimulated, respectively) to analyze glucose, insulin, and C-peptide levels.

The inclusion criteria of this study were as follows: aged 19 years or older, newly diagnosed with T2D according to the 2019 guidelines of the Korean Diabetes Association, serum βHB levels measured at the center since its availability in 2017, TE results measured within six months before or after the baseline measurement of βHB. Patients who had received organ transplantation or chemotherapy, as well as those who used steroids or had previously taken antidiabetic medications before the initial blood sampling or visited the emergency room due to hyperglycemia, were excluded. Additionally, we excluded those who were extreme outliers [i.e., alanine aminotransferase (ALT) ≥1,000 IU/L, triglycerides (TGs) ≥1,000 mg/dL], aged ≥80 years, and with glycated hemoglobin (HbA1c) ≥11% from the study. Finally, 361 newly diagnosed T2D patients were included as study participants. The study protocol was approved by the institutional review board of Severance Hospital (4–2022–1101). Informed consent was waived because of the retrospective nature of the study.

### Clinical measurement and laboratory assessment

Patient data, such as age, sex, weight, height, and use of antidiabetic drugs or antihypertensive drugs at baseline, were collected by reviewing electronic medical records. The following laboratory variables were measured at baseline: serum HbA1c, creatinine, aspartate aminotransferase (AST), ALT, total cholesterol, high-density lipoprotein cholesterol (HDL-C), low-density lipoprotein cholesterol (LDL-C), TGs, urine albumin, urine creatinine, and serum βHB. Body mass index (BMI) was calculated as body weight divided by height squared (kg/m^2^) and the estimated glomerular filtration rate (eGFR) was assessed based on the four-variable modification of diet in the renal disease study formula. Glucometabolic parameters, including serum glucose, C-peptide, and insulin, were measured after overnight fasting and postprandial. The postprandial level was obtained 90 minutes after the mixed-meal test (Mediwell Diabetic Meal; Meail Dairies Co., Yeongdong-gun, Chungbuk, Korea) was given. The homeostasis model assessments of insulin resistance (HOMA-IR) index and HOMA-β were calculated to assess insulin resistance and pancreatic β-cell function.

The ketogenic capacity of the subject was assessed prior to the administration of diabetes medication by measuring the concentration of serum βHB, which is the most prevalent form of ketone bodies. The concentration of serum βHB in a fasting state was quantified with a commercial enzymatic assay from Landox Laboratories Ltd. (County Antrim, UK) and an Atellical CH 930 analyzer (Siemens Healthcare Diagnostics, Marburg, Germany). In cases where the concentration of βHB was below the lower limit of detection for the assay, a value of zero was recorded.

### Measurements of hepatic steatosis using transient elastography

TE was performed by an experienced specialist without knowing the subject’s clinical details using a Fibroscan 501^®^ (Echosens, Paris, France). The procedure was conducted with a standard probe and the final value was obtained using a standard procedure. The principles of controlled attenuation parameter (CAP) measurement for TE have been described previously (19, 20). Hepatic steatosis was categorized into four grades based on the CAP value [decibels per meter (dB/m)]: less than 238 dB/m for steatosis stage 0 (S0), 238 dB/m to 259 dB/m for steatosis stage 1 (S1, mild), 260 dB/m to 292 dB/m for steatosis stage 2 (S2, moderate), and greater than 293 dB/m for steatosis stage 3 (S3, severe) (21, 22).

### Statistical analysis

The characteristics of the study participants were analyzed according to the status of ketogenesis using Student’s *t*-test for continuous variables and Pearson’s χ^2^ test for categorical variables. All continuous variables were presented as the mean ± standard deviation, and categorical variables were presented as a number with percentage (%). The correlations between serum βHB levels and steatosis stages and CAP scores were assessed with Pearson’s correlation analysis. Multivariable logistic regression analysis was applied to determine the independent association between ketogenesis and hepatic steatosis. The ketogenic capacity defined as intact or impaired ketogenesis based on the median serum βHB level was used as a variable in the logistic regression. Age, sex, BMI, HbA1c, LDL-C, HOMA-IR, and HOMA-β were adjusted in the multivariable regression analysis. Statistical analyses were performed using R software version 3.6.3 (R Project for Statistical Computing, Vienna, Austria). The adjusted odds ratio (OR) and 95% confidence interval (CI) were determined. P-values <0.05 were considered statistically significant.

## Results

### Clinical and laboratory characteristics of participants

A total of 361 newly diagnosed T2D patients who satisfied both inclusion and exclusion criteria were included as study participants. Subjects were divided into intact (βHB>0, n=163) and impaired ketogenesis (βHB=0, n=198) groups according to the baseline level of βHB. Baseline characteristics stratified by the level of βHB are presented in **Table 1**. The mean age and BMI of the study subjects were 54.3 ± 12.5 years and 27.4 ± 4.2 kg/m^2^, respectively. A total of 60.9% of the patients were men. Both groups had similar characteristics in terms of age, sex, BMI, and liver enzyme levels. However, the group with intact ketogenesis had notably higher levels of HbA1c, indicating poorer glycemic control, compared to the group with impaired ketogenesis. In the context of insulin secretory function, the intact ketogenesis group also demonstrated markedly lower levels of fasting and postprandial C-peptide and insulin levels compared to patients with impaired ketogenesis. Additionally, subjects in the intact ketogenesis group had decreased HOMA-β and lower HOMA-IR without significance compared to the impaired group. Meanwhile, TG levels were lower and LDL-C levels were higher, without statistical significance, but TG/LCL-C was significantly lower in the intact group. Collectively, the subjects with intact ketogenesis capacity at the time of first diagnosis of T2D showed higher serum glucose levels with more prominent insulin deficiency and potentially lower insulin resistance.

**Table 1:** Characteristics of the study population according to baseline βHB.

		Intact ketogenesis (N=163)	Impaired ketogenesis (N=163)	*P*-value
Demographic				
Age (years)		51.8 ± 13.6	55.2 ± 11.9	0.116
Sex (Male, n (%))		100 (61.3%)	120 (60.6%)	0.971
HTN		82 (50.3%)	114 (57.6%)	0.203
BMI (kg/m^2^)		27.1 ± 4.2	27.7 ± 4.1	0.122
Biochemistry				
AST (IU/L)		40.9 ± 39.3	36.0 ± 22.2	0.154
ALT (IU/L)		47.4 ± 44.4	45.3 ± 43.6	0.646
Total cholesterol (mg/dL)		189.2 ± 48.2	187.5 ± 46.0	0.724
TG (mg/dL)		169.7 ± 130.8	181.0 ± 114.5	0.380
HDL-C (mg/dL)		47.5 ± 13.4	46.4 ± 11.0	0.405
LDL-C (mg/dL)		114.0 ± 41.1	108.7 ± 42.9	0.241
TG/LDL-C		1.5 ± 1.2	2.1 ± 3.5	0.016
eGFR (ml/min/1.73 m²)		89.6 ± 19.1	91.0 ± 24.4	0.753
uACR (mg/g creatinine)		50.4 ± 147.0	57.6 ± 171.6	0.678
Gluco-metabolic parameters				
Fasting glucose (mg/dL)		148.8 ± 50.1	144.3 ± 59.6	0.436
** HbA1c (%)**		**8.0 ± 1.3**	**7.5 ± 1.0**	**0.001**
** Fasting C-peptide (ng/mL)**		**2.8 ± 1.1**	**3.2 ± 1.2**	**<0.001**
** Postprandial C-peptide (ng/mL)**		**6.6 ± 2.6**	**7.3 ± 2.7**	**0.027**
** Fasting insulin (μIU/mL)**		**12.9 ± 9.5**	**16.7 ± 21.4**	**0.019**
** Postprandial insulin (μIU/mL)**		**61.3 ± 42.4**	**74.7 ± 54.7**	**0.010**
HOMA-IR		5.1 ± 5.8	6.0 ± 8.5	0.240
** HOMA-β**		**31.6 ± 21.3**	**44.2 ± 51.0**	**0.002**
**βHB (mmol/L)**		**0.2 ± 0.2**	**0.0 ± 0.0**	**<0.001**
**βHB x 100/glucose**		**0.2 ± 0.3**	**0.0 ± 0.0**	**<0.001**
Fibroscan				
Steatosis	0	29 (17.8%)	22 (11.1%)	0.090
	1	24 (14.7%)	19 (9.6%)	
	2	33 (20.2%)	48 (24.2%)	
	3	77 (47.2%)	109 (55.1%)	
**Steatosis (CAP ≥ 260)**	**0,1**	**53 (32.5%)**	**41 (20.7%)**	**0.015**
	**2,3**	**110 (67.5%)**	**157 (79.3%)**	
CAP (dB/m)		290.1 ± 52.0	295.5 ± 43.0	0.287

BMI, body mass index; HbA1c, glycosylated hemoglobin; TG, triglycerides; HDL-C, high-density lipoprotein cholesterol; LDL-C, low-density lipoprotein cholesterol; HOMA-IR, homeostasis model assessment for insulin resistance; HOMA- β, homeostasis model assessment of beta cell function; βHB, β-hydroxybutyrate; AST, aspartate aminotransferase; ALT, alanine aminotransferase; HTN, hypertension; eGFR, estimated glomerular filtration rate; uACR, urine albumin-creatinine ratio.

Bold values ​​indicate statistically significant values.

In this study, the presence of significant MAFLD was defined as steatosis stage ≥S2 (moderate–severe) (21, 22). The intact ketogenesis group showed a tendency to lower levels of steatosis stage and CAP values (290.1 ± 52.0 vs. 295.5 ± 43.0) compared with the impaired ketogenesis group, but this difference was not statistically significant. However, moderate–severe hepatic steatosis was less common in the intact group (67.5% vs. 79.3%) with statistical significance (*p*=0.015).

### Intact ketogenesis is correlated with lower CAP value

Collinearity among ketogenic capacities based on serum βHB levels and hepatic steatosis was calculated using Pearson’s correlation analysis (**Table 2**). Liver steatosis stages stratified from stage S0 to S4 showed a significant negative correlation with βHB and moderate–severe hepatic steatosis showed a negative correlation. Additionally, a negative correlation was observed between CAP scores and βHB levels (*r*=-0.094, *p*=0.009).

**Table 2:** Correlations between βHB and hepatic steatosis.

	In all subjects (N=361)	
	β-hydroxybutyrate	
	*r*	p-value
**Steatosis (Stage 0–3)**	**-0.155**	**0.003**
**Moderate to severe steatosis (Stage 2–3)**	**-0.129**	**0.015**
**CAP (dB.m)**	**-0.094**	**0.009**

CAP, controlled attenuation parameter.

Bold values ​​indicate statistically significant values.

### Ketogenic capacity is associated with MAFLD

**Figure 1:**
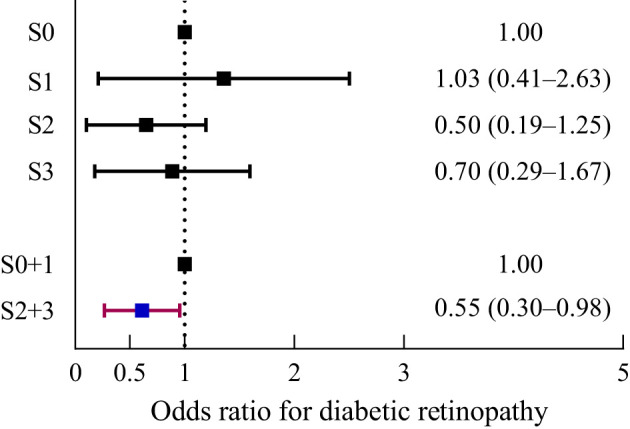
Risk of hepatic steatosis according to intact and impaired ketogenesis.

To evaluate whether intact ketogenesis was independently associated with hepatic steatosis, a multiple logistic regression analysis was performed adjusting for age, sex, BMI, HbA1c, LDL-C, HOMA-IR, and HOMA-β. When analyzing each steatosis stage separately, no significant association was found between the ketogenic capacity and all stages of hepatic steatosis. However, statistically significant results were obtained when comparing the stages of moderate–severe MAFLD with normal–mild MAFLD (**Figure 1**). Intact ketogenesis was associated with a lower OR for the risk of higher hepatic steatosis stage (adjusted OR, 0.55; 95% CI, 0.30–0.98). Adjusted ORs for each variable of hepatic steatosis are summarized in **Table 3**. BMI (adjusted OR, 1.43; 95% CI, 1.28–1.61; *p*<0.001) and HbA1c (adjusted OR, 1.37; 95% CI, 1.02–1.85; *p*=0.037) were associated with moderate–severe hepatic steatosis after adjusting for multiple risk factors. Intact ketogenesis was notably associated with a lower risk of moderate–severe hepatic steatosis compared with impaired ketogenesis.

**Table 3:** Logistic regression analysis for MAFLD incidence based on TE.

In all subjects (N=361)	Hepatic steatosis	
	Adjusted OR (95% CI)	P-value
Age (years)	0.98 (0.95-1.01)	0.147
Sex (female vs. male)	0.66 (0.36-1.20)	0.178
BMI (kg/m2)	**1.43 (1.28-1.61)**	**<0.001**
HbA1c (%)	**1.37 (1.02-1.85)**	**0.037**
LDL-C (mg/dL)	1.01 (1.00-1.01)	0.119
HOMA-IR	0.95 (0.87-1.08)	0.277
HOMA-β	1.02 (0.99-1.04)	0.089
Ketogenic capacity*	**0.55 (0.30-0.98)**	**0.044**

BMI, body mass index; HbA1c, glycosylated hemoglobin; LDL-C, low-density lipoprotein cholesterol; HOMA-IR, homeostasis model assessment for insulin resistance; HOMA-β, homeostasis model assessment of beta cell function.

*Ketogenic capacity is defined as being divided into intact/impaired based on the median serum βHB level. The bolded items are statistically significant variables.

## Discussion

Most studies have reported reduced risk for incident hepatic steatosis or advanced hepatic fibrosis in subjects with fasting ketonuria and intact ketonemia (23, 24); however, some studies reported higher ketone levels in subjects with suspected fatty liver disease, prediabetes, or diabetes (25–27). In this cross-sectional study, we investigated the relationship between ketogenic capacity and MAFLD in newly diagnosed T2D patients by measuring serum βHB and CAP levels using TE to assess the ketogenic capacity and MAFLD, respectively. We used the CAP score rather than liver stiffness measurements (LSMs) because we expected that metabolic properties, such as ketogenesis, would preferentially appear in the form of hepatic steatosis rather than fibrosis.

This study had three main findings. First, 42.7% of the T2D patients had serum βHB>0.1 mmol/L, and 3.3% of the patients had >0.57 mmol/L, which was the median serum βHB level of subjects with 2+ ketonuria in the previous study (28). Second, intact ketogenesis predicted a lower risk of moderate–severe MAFLD assessed by TE. Third, T2D patients with intact ketogenesis tended to be more insulin sensitive and had relatively lower insulin secretory function compared to those with impaired ketogenesis.

Either physiologic or pathologic ketogenesis in humans is an effective way to supply efficient energy because it has an adaptive mechanism that allows the liver to utilize deposited TGs to provide energy in the form of ketone bodies during periods of glucose deprivation as well as to dispose of the delivered FAs in the liver; delivered FAs are converted to acetyl-CoA through β-oxidation, thereby undergoing non-oxidative pathway processing to produce ketone bodies (6, 29). In the excess glucose status of T2D, activation of ketogenesis might not be a pathophysiologic mechanism for producing more metabolic fuel but may be an efficient pathway for the disposal of excess FAs. Impairment of the ketogenic pathway in T2D might be activation of gluconeogenesis rather than ketone body generation in the dysregulated liver. In a dysregulated situation, converted acetyl-CoA from FAs is oxidized to carbon dioxide via the TCA cycle, which increases hepatic oxygen consumption and promotes gluconeogenesis (30). As a result, both hepatic *de novo* lipogenesis and oxidative stress are increased, thereby inducing or aggravating MAFLD. In addition, intact or efficient ketogenesis might have other putative mechanisms that prevent the development of MAFLD; the ketogenic process is reported to induce the hepatic peroxisome proliferation-activated receptor α (PPARα)-fibroblast growth factor 21 (FGF21) axis, which plays a critical role in energy metabolism and whose dysregulation is associated with MAFLD (31, 32). In addition, the ketogenic pathway activates hepatic autophagy and suppresses inflammatory responses (33–35), which is also thought to be beneficial in improving MAFLD.

With respect to intact ketogenesis and MAFLD, our measurements in βHB are in line with a previous study that obtained serum βHB measurements in T2D patients using a nuclear magnetic resonance spectroscopy-based test (36) and found reduced risk for incident hepatic steatosis or advanced hepatic fibrosis in subjects with fasting ketonuria (18, 23, 24). In this study, T2D patients with intact ketogenesis showed a tendency towards lower steatosis stages and CAP values. In particular, the prevalence of moderate–severe hepatic steatosis was significantly lower in the intact ketogenesis group compared to the impaired group. In the logistic regression analysis, intact ketogenesis was significantly associated with lower risk of moderate–severe steatosis. These results are in line with our previous study that reported the relationship between ketogenic capacity and hepatic steatosis indices (18). Considering the ketogenic mechanisms that were described above, the results of our study, showing an association between intact ketogenesis and lower risk of moderate–severe MAFLD in subjects with T2D, are convincing.

Relatively severe insulin resistance in impaired ketogenic subjects is considered to have contributed to the development or aggravation of MAFLD. The greater difference in the plasma insulin levels between the two groups, compared to the difference in the HOMA-IR, might be due to lower hepatic insulin clearance in the impaired ketogenesis group, which has been reported to be associated with MAFLD (37).

The strength of this study, to the best of our knowledge, is it is the first to show the association between ketogenic capacity and hepatic steatosis using TE imaging in subjects with drug-naïve, newly diagnosed T2D. The present study has some limitations to be considered. First, our study was a cross-sectional design, so we cannot offer a conclusive opinion on the association between ketogenic capacity and MAFLD. Second, we used TE to investigate the presence and extent of MAFLD. Although it is relatively less accurate than magnetic resonance imaging-based methods (38, 39), TE is well known for its good accessibility, reproducibility, and validation. Therefore, TE is recommended as an acceptable non-invasive method to assess MAFLD according to current guidelines (40, 41). Third, we only used blood βHB to assess ketogenic capacity. As mentioned in the introduction section, ketone bodies include βHB, acetoacetate, and acetone. Although βHB is the most abundant form of the blood ketone bodies, lack of data on acetoacetate and acetone might be a limitation. Especially, the βHB/acetoacetate ratio is known to reflect the hepatic redox state, which is a major phenotype of the genetic aspect of MAFLD pathogenesis (42). Therefore, if we had collected data on acetoacetate, the mechanistic explanation might be a little clearer. Finally, we do not have histological data based on liver biopsies, the gold standard for diagnosis of liver steatohepatitis and fibrosis.

## Conclusion

In T2D patients, intact ketogenesis is related to the lower risk of moderate–severe MAFLD assessed by TE. This result is in line with a previous study that used MAFLD indices and is supported by putative pathophysiologic mechanisms, such as the favorable aspects of *de novo* lipogenesis and oxidative stress, induction of the PPARα-FGF21 axis, activation of hepatic autophagy, and suppression of inflammatory processes. Longitudinal studies including large populations, advanced imaging modalities, and/or histological data are needed to elucidate the relationship between ketogenesis and MAFLD.

## Data availability statement

The original contributions presented in the study are included in the article/supplementary material. Further inquiries can be directed to the corresponding author.

## Ethics statement

The studies involving human participants were reviewed and approved by the Institutional review board of Severance Hospital. Written informed consent for participation was not required for this study in accordance with the national legislation and the institutional requirements.

## Author contributions

SL: Conceptualization, Data curation, Writing – original draft. JB: Conceptualization, Data curation, Formal Analysis, Writing – original draft. SK: Data curation, Writing – review & editing. ML: Conceptualization, Writing – review & editing. Y-HL: Conceptualization, Writing – review & editing. EK: Conceptualization, Writing – review & editing. B-SC: Conceptualization, Writing – review & editing. B-WL: Conceptualization, Data curation, Formal Analysis, Methodology, Writing – review & editing.
